# The cytoprotective role of DJ-1 and p45 NFE2 against human primary alveolar type II cell injury and emphysema

**DOI:** 10.1038/s41598-018-21790-3

**Published:** 2018-02-23

**Authors:** Li Hui Tan, Karim Bahmed, Chih-Ru Lin, Nathaniel Marchetti, Sudhir Bolla, Gerard J. Criner, Steven Kelsen, Muniswamy Madesh, Beata Kosmider

**Affiliations:** 1 Department of Thoracic Medicine and Surgery, Temple University, Philadelphia, PA 19140 United States; 2 Center for Inflammation, Translational and Clinical Lung Research, Temple University, Philadelphia, PA 19140 United States; 3 Medical Genetics and Molecular Biochemistry, Temple University, Philadelphia, PA 19140 United States; 4 Department of Physiology, Temple University, Philadelphia, PA 19140 United States

## Abstract

Emphysema is characterized by irreversibly enlarged airspaces and destruction of alveolar walls. One of the factors contributing to this disease pathogenesis is an elevation in extracellular matrix (ECM) degradation in the lung. Alveolar type II (ATII) cells produce and secrete pulmonary surfactants and proliferate to restore the epithelium after damage. We isolated ATII cells from control non-smokers, smokers and patients with emphysema to determine the role of NFE2 (nuclear factor, erythroid-derived 2). NFE2 is a heterodimer composed of two subunits, a 45 kDa (p45 NFE2) and 18 kDa (p18 NFE2) polypeptides. Low expression of p45 NFE2 in patients with emphysema correlated with a high ECM degradation. Moreover, we found that NFE2 knockdown increased cell death induced by cigarette smoke extract. We also studied the cross talk between p45 NFE2 and DJ-1. DJ-1 protein is a redox-sensitive chaperone that protects cells from oxidative stress. We detected that cigarette smoke significantly increased p45 NFE2 levels in DJ-1 KO mice compared to wild-type mice. Our results indicate that p45 NFE2 expression is induced by exposure to cigarette smoke, has a cytoprotective activity against cell injury, and its downregulation in human primary ATII cells may contribute to emphysema pathogenesis.

## Introduction

Chronic obstructive pulmonary disease (COPD) is the fourth cause of death in the world and it is currently presenting a major global public health challenge^1^. It may become the fifth ranked cause of disability, affecting approximately 10% of people over 45 years^2,3^. It is estimated that approximately 14 million people in the United States have this disease^4^. Cigarette smoke (CS) is the key source of reactive oxygen species (ROS) and the main risk factor of COPD development. There are clearly different clinical phenotypes of COPD, which also encompasses emphysema. Pulmonary emphysema is characterized by an irreversibly increased airspaces and destruction of alveolar walls. One of the factors contributing to this disease pathogenesis is an elevation in extracellular matrix (ECM) degradation in the lung^5,6^. ROS can fragment ECM components^7^, which is an important factor in triggering lung inflammation and causing subsequent airspace enlargement^8^. It has been reported that the composition of the ECM varies among non-smokers, smokers and COPD patients^5,9^. There is an increased activity of metalloproteinases (MMPs) in lung tissue in individuals with this disease. MMPs are proteolytic enzymes that degrade ECM components and play a role in initiating and maintaining inflammation after exposure to CS and in emphysema^6^. MMPs expression can be modulated and activated by a cell surface protein CD147^10^, which is an extracellular matrix metalloproteinase inducer (EMMPRIN). Cathepsin B belongs to cysteine proteases^11,12^ and its levels were increased in cells treated with CS^13^. Moreover, intratracheal instillation of cathepsin B induced emphysema in hamsters^14^. It can also increase the activity of MMPs by destroying their inhibitors^15^ and contribute to alveolar wall destruction^3,16^.

Alveolar type II (ATII) cells produce and secrete pulmonary surfactants, which reduce the surface tension at the air/liquid interface within the alveoli of the lung. ATII cells proliferate and differentiate into alveolar type I (ATI) cells to restore the epithelium after damage^17^. ROS generation and elevated oxidative stress induced by CS cause alveolar epithelial cell death^18^. We have also reported human and murine primary ATII and ATI-like cell injury induced by CS *in vitro* and *in vivo*^19–23^. Recent findings indicate decreased ATII cell proliferation and transdifferentation to ATI cells in patients with COPD^24^. Moreover, it has been reported that ATII cell apoptosis contributes to emphysema development^24^. However, the mechanism of proteinase expression in ATII cells as a result of dysregulated antioxidant signalling, which may contribute to this disease development is not well known.

We focused on the role of a nuclear factor, erythroid-derived 2 (NFE2) in ATII cells isolated from patients with emphysema and control non-smokers and smokers. NFE2 is a heterodimer composed of two subunits, a 45 kDa (p45 NFE2) and 18 kDa (p18 NFE2) polypeptides^25^. Transcriptional activation capacity of NFE2 depends on the N-terminal region of p45^26^. Nuclear erythroid 2-related factor-2 (NRF2) is a key regulator of antioxidant defense system and activates the transcription of numerous cytoprotective genes to eliminate ROS^25,27^. Since p45 NFE2 and NRF2 belong to Cap′N′Collar (CNC) family^25^ and possess similar DNA-binding specificities, we hypothesized that p45 NFE2 will have a cytoprotective activity against ATII cell injury by CS. We have previously reported that NRF2 expression in human primary ATII cells exposed to CS is modulated by DJ-1^19^. DJ-1 protein is a redox-sensitive chaperone that protects cells from oxidative stress^28^. We have recently showed that DJ-1 overexpression has a cytoprotective effect against primary ATII cell injury induced by cigarette smoke extract and was able to restore the impaired antioxidant defense system in these cells isolated from heavy smokers^19^. Therefore, we also wanted to analyze the cross talk between p45 NFE2 and DJ-1 to determine their functional relationship in ATII cells under oxidative stress induced by CS and in emphysema. To our knowledge, this is the first study on the role of p45 NFE2 and DJ-1 in human ATII cells in this disease.

## Results

### High MMP9, CD147 and cathepsin B expression in human ATII cells in emphysema

**Figure 1:**
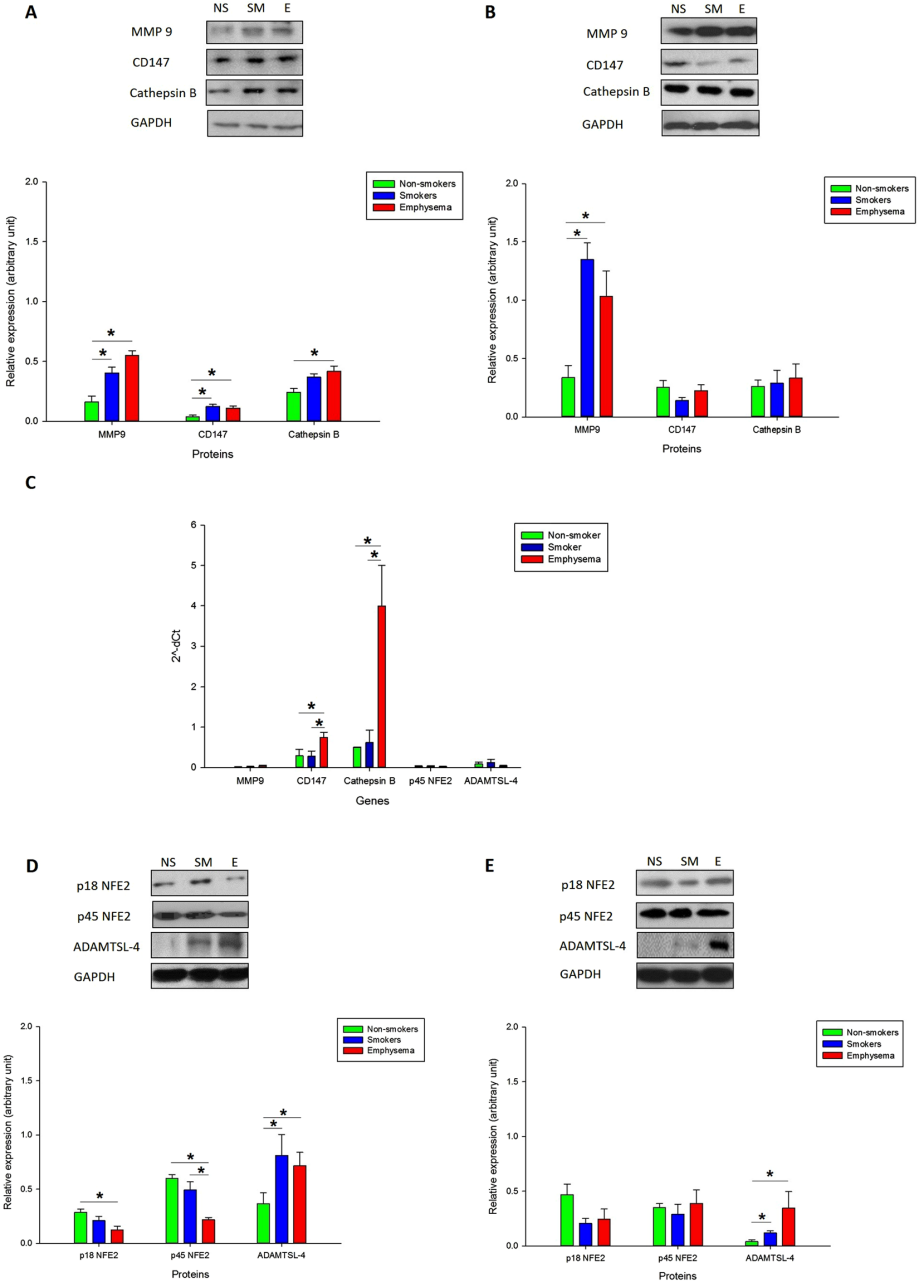
MMP9, CD147, cathepsin B, p18 NFE2, p45 NFE2 and ADAMTSL-4 expression in ATII cells and lung tissue obtained from non-smokers (NS), smokers (SM) and patients with emphysema (E). MMP9, CD147 and cathepsin B expression in freshly isolated ATII cells (**A**) and lung tissue (**B**) as detected by Western blotting analysis. (**C**) MMP9, CD147, cathepsin B, p45 NFE2 and ADAMTSL-4 mRNA levels in lung tissue by RT-PCR. (**D,E**) p18 NFE2, p45 NFE2 and ADAMTSL-4 expression in freshly isolated ATII cells (**D**) and lung tissue (**E**). Protein levels were analyzed by Western blotting. Densitometric analysis is also shown. *Statistically significant difference (*p* < 0.05) is shown for comparison between non-smokers and smokers, between smokers and emphysema patients and between non-smokers and emphysema patients. Data are shown as the mean (±s.e.m.).

Considering the role of MMP9, CD147 and cathepsin B in COPD development^6,10,12^, we wanted to check their intracellular levels in ATII cells and lung tissue obtained from patients with emphysema compared to control non-smokers and smokers. We found statistically significant increase in MMP9, CD147 and cathepsin B levels in ATII cells isolated from individuals with this disease compared to non-smokers by Western blotting (Fig. 1A). We also detected higher MMP9 expression in lung tissue obtained from these patients in comparison with controls (Fig. 1B). Increased MMP9, CD147 and cathepsin B levels in ATII cells in emphysema may suggest their contribution to alveolar wall destruction.

We also checked MMP9, CD147 and cathepsin B mRNA levels by RT-PCR in lung tissue obtained from control non-smokers, smokers and patients with emphysema (Fig. 1C). We found significantly higher expression of CD147 and cathepsin B in patients with this disease, suggesting its regulation at the transcription level. However, we did not detect any significant difference in MMP9 mRNA expression between these groups. Our results suggest that MMP9 may be regulated at the protein level in emphysema.

### Downregulated p45 NFE2 expression in human ATII cells in emphysema

NFE2 is composed of two subunits p18 and p45 and may be involved in the antioxidant defense system^25^. We found significantly lower levels of p18 NFE2 and p45 NFE2 in ATII cells in emphysema compared to control non-smokers and smokers by Western blotting (Fig. 1D). However, we did not detect significant changes in their expression in lung tissue (Fig. 1E), which may be related to the presence of various cell types in these samples. Moreover, we analyzed p45 NFE2 mRNA expression by RT-PCR in lung tissue (Fig. 1C) and ATII cells **(**Supplementary Fig. S1) and did not detect changes at gene levels in emphysema compared to controls. This suggests the p45 NFE2 regulation at the posttranslational level in emphysema. We also checked expression of ADAMTSL-4, which is a member of ADAMTS (a disintegrin and metalloproteinase with thrombospondin motif-like) family^29^ and has two specific DNA binding sites for a NFE2 transcription factor. We found significantly increased ADAMTSL-4 expression in ATII cells (Fig. 1D) and lung tissue (Fig. 1E) in emphysema in comparison with non-smokers.

**Figure 2:**
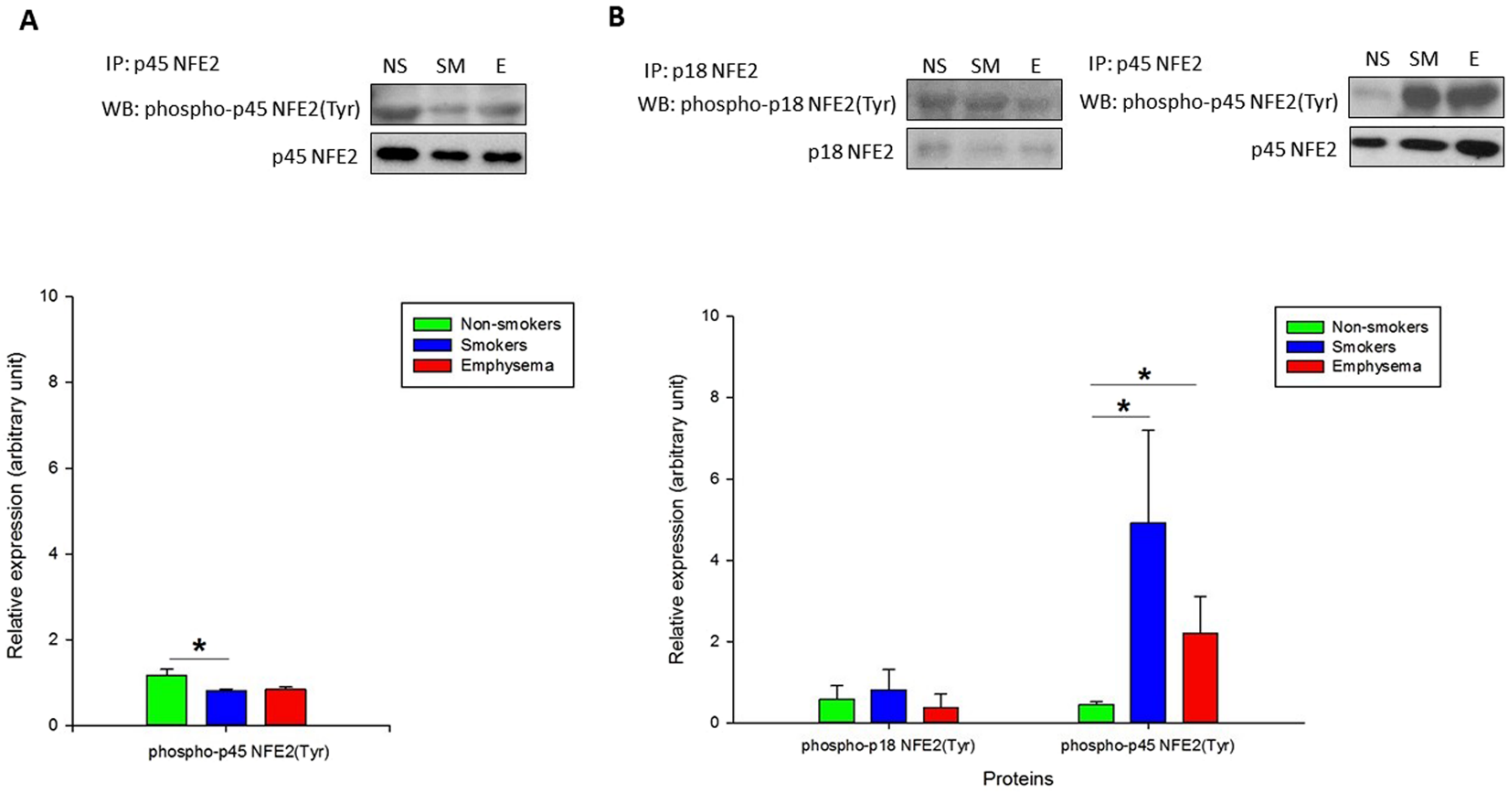
p45 NFE2 and p18 NFE2 tyrosine phosphorylation in human ATII cells and lung tissue obtained from control non-smokers (NS) and smokers (SM) and patients with emphysema (E). Immunoprecipitation was performed in freshly isolated ATII cells (**A**) and lung tissue (**B**) followed by Western blotting analysis. Densitometric quantification is also shown. *Statistically significant difference (*p* < 0.05). Data are shown as the mean (±s.e.m.).

Next we wanted to further determine the regulation of NFE2 expression at the protein level. We analyzed phosphorylation of tyrosine, serine and threonine residues within p45 NFE2 in ATII cells (Fig. 2A) and lung tissue (Fig. 2B) obtained from non-smokers, smokers and emphysema patients using immunoprecipitation followed by Western blotting. We found decreased tyrosine phosphorylation within p45 NFE2 in ATII cells in smokers. However, we observed its significantly high levels in lung tissue in smokers and emphysema patients in comparison with non-smokers. We also checked tyrosine phosphorylation within p18 NFE2, however, we didn’t detect significant changes. Moreover, we analyzed serine and threonine phosphorylation within p45 NFE2 in lung tissue and we didn’t observe differences in their levels between these three groups (Supplementary Fig. S2). The discrepancy between our results obtained from ATII cells and lung tissue may have been caused by the presence of various cell types in latter samples.

### P45 NFE2 has a cytoprotective activity

**Figure 3:**
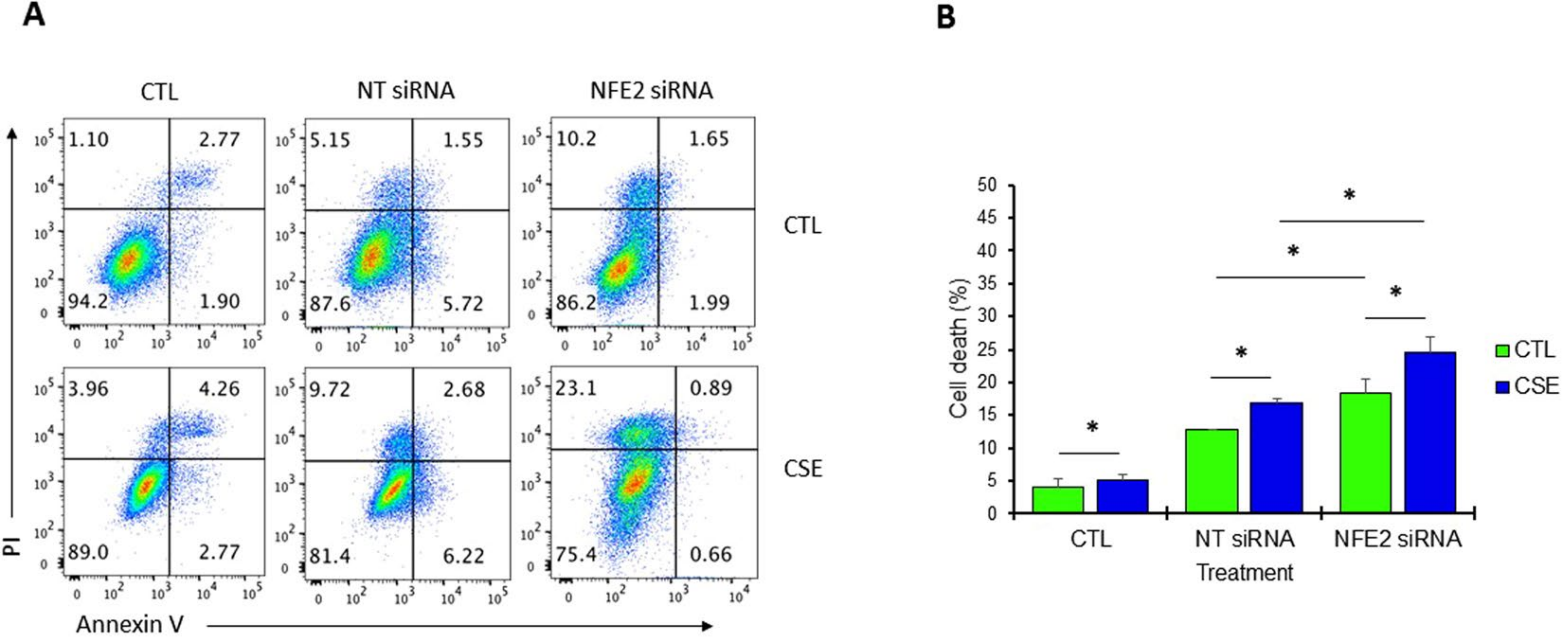
NFE2 knockdown increases cell death induced by CSE in A549 cells *in vitro*. (**A**) A549 cells were transfected with 100 nM NFE2 siRNA or non-targeting (NT) siRNA followed by exposure to CSE for 24 h. Representative flow cytometry images using Annexin V and PI staining are shown. (**B**) NFE2 knockdown significantly increased cell death after treatment with CSE compared to control. *Statistically significant difference (*p* < 0.05). Data are shown as the mean (±s.e.m.).

We wanted to further determine the function of p45 NFE2 in cells. We knocked down NFE2 in A549 cells using siRNA strategy (Supplementary Fig. S3) followed by treatment with CSE. Cell death was analyzed by staining with Annexin V and PI (Fig. 3A). We found significantly higher cell death in A549 cells with NFE2 knockdown followed by exposure to CSE compared to control (Fig. 3B). Our results indicate that NFE2 knockdown sensitizes A549 cells to injury induced by CSE.

### P45 NFE2 interacts with DJ-1 in human primary ATII cells

**Figure 4:**
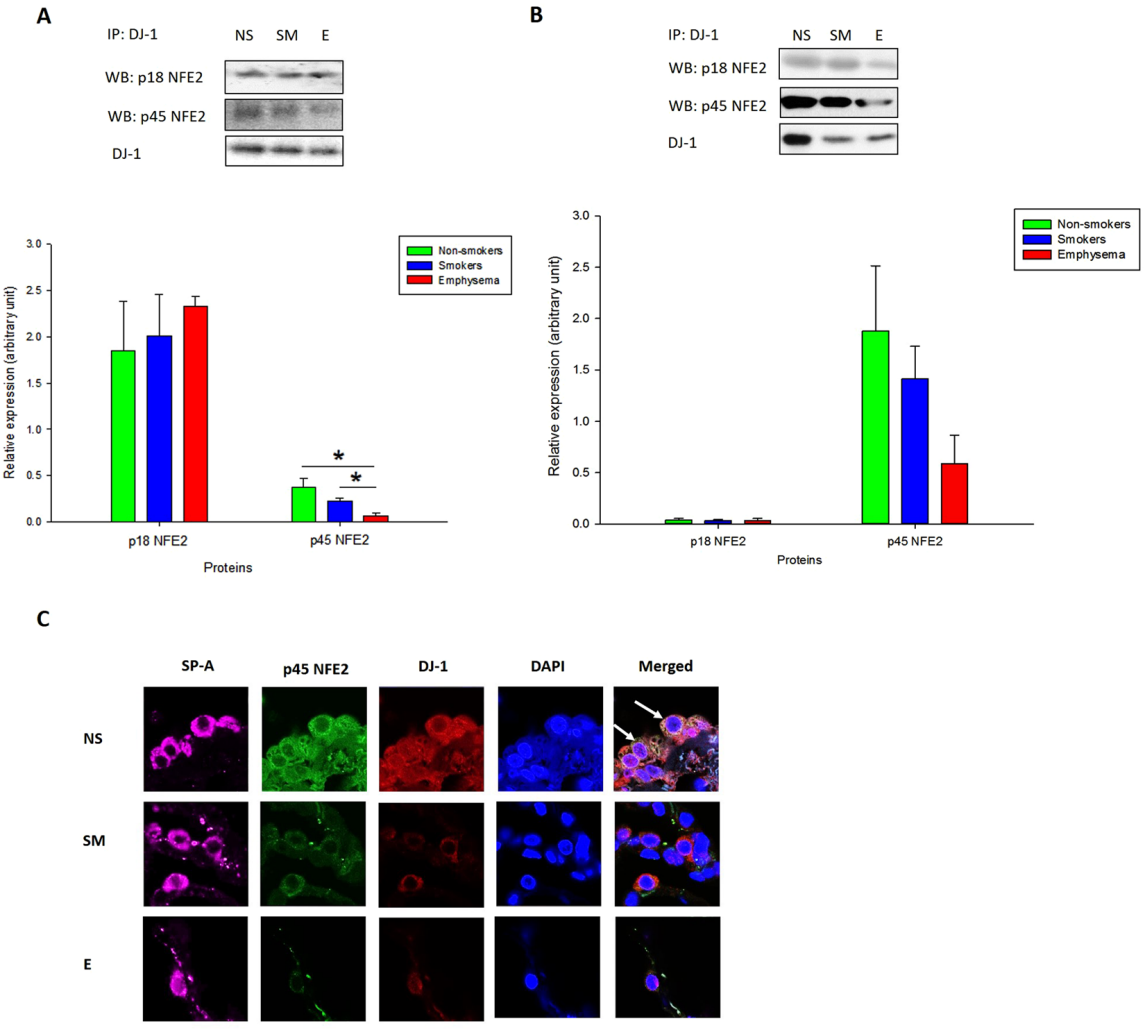
p45 NFE2 interaction with DJ-1 in ATII cells and lung tissue obtained from non-smokers (NS), smokers (SM) and patients with emphysema (E). DJ-1 was co-immunoprecipitated in freshly isolated ATII cells (**A**) or lung tissue (**B**) followed by Western blotting analysis to determine its interaction with p45 NFE2 or p18 NFE2 as described in Materials and Methods. Relative expression is also shown. (**C**) p45 NFE2 (green) and DJ-1 (red) expression in ATII cells identified using SP-A staining (violet) in lung tissue by immunohistofluorescence. Cell nuclei were stained with DAPI (blue). The strongest co-localization of p45 NFE2 and DJ-1 is indicated by white arrows. *Statistically significant difference (*p* < 0.05). Data are shown as the mean (±s.e.m.).

It has been reported that DJ-1 has a cytoprotective activity^28^. We have recently showed that it also modulates the antioxidant defense system regulated by NRF2^19^. Therefore, here we wanted to determine the association between DJ-1 and p45 NFE2. We immunoprecipitated DJ-1 followed by Western blotting analysis and determined p18 NFE2 and p45 NFE2 expression in lung tissue and ATII cells obtained from control non-smokers, smokers or patients with emphysema. We did not detect significant p18 NFE2 and DJ-1 interaction in ATII cells and lung tissue (Fig. 4A,B; Supplementary Fig. S4). However, we observed significantly lower p45 NFE2 and DJ-1 interaction in ATII cells isolated from patients with emphysema compared to control non-smokers or smokers. We also used immunohistofluorescence to determine p45 NFE2 and DJ-1 expression in ATII cells identified by SP-A staining (Fig. 4C). First, we found cytoplasmic co-localization of p45 NFE2 and DJ-1. Second, we detected their lower expression and co-localization in ATII cells in emphysema compared to controls, which correlates with our results obtained by immunoprecipitation and Western blotting. Our results suggest that low p45 NFE2 and DJ-1 interaction in ATII cells in emphysema may contribute to decreased antioxidant defense system in this disease. We confirmed our findings by assessing NRF2 levels and observed its lowest expression in these patients (Supplementary Fig. S5). We selected NRF2, since first, it is involved in the antioxidant defense system^27^, second, we have previously shown that DJ-1 modulates NRF2 levels^19^, and third, NRF2 and p45 NFE2 belong to Cap’N’Collar (CNC) family^25^.

### P45 NFE2 expression in DJ-1 KO mice exposed to CS

**Figure 5:**
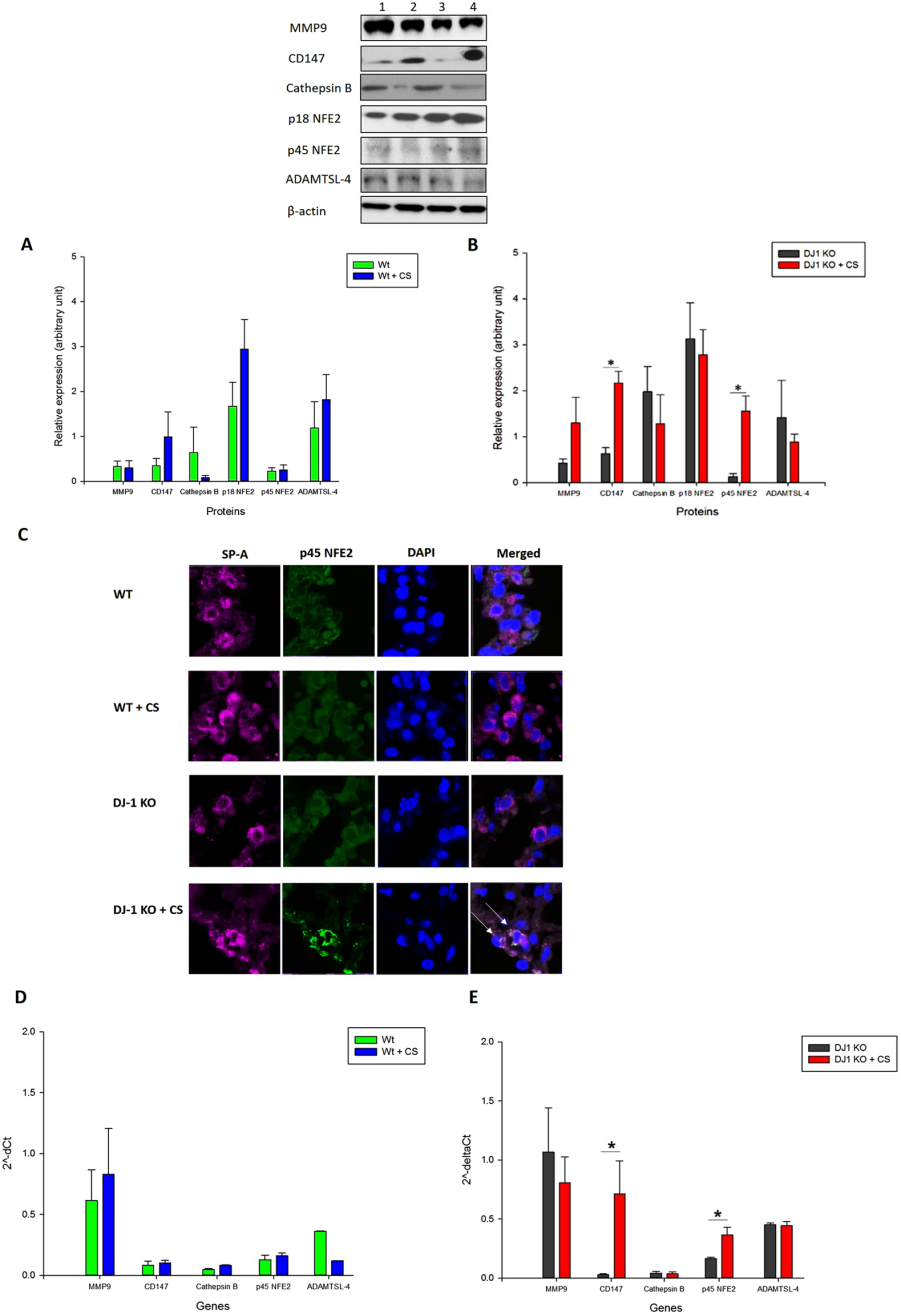
MMP9, CD147, cathepsin B, p18 NFE2, p45 NFE2 and ADAMTSL-4 expression in wild-type and DJ-1 KO mice. Wild-type (WT) (**A**) and DJ-1 KO mice (**B**) were exposed to 150 mg/m^3^ cigarette smoke (CS) for 2 h/day for 3 weeks as described in Materials and Methods section. Protein levels were analyzed in lung tissue by Western blotting. Lane 1 – WT mice, Lane 2 – WT + CS, Lane 3 – DJ-1 KO mice, Lane 4 – DJ-1 KO mice + CS. Relative expression is also shown. (**C**) p45 NFE2 expression (green) in ATII cells identified using SP-A antibody (violet) in lung tissue obtained from wild-type and DJ-1 KO mice by immunohistofluorescence. Cell nuclei were stained with DAPI (blue). (**D**,**E**) MMP9, CD147, cathepsin B, p45 NFE2 and ADAMTSL-4 mRNA expression in lung tissue from wild-type (**D**) and DJ-1 KO (**E**) mice by RT-PCR. The strongest p45 NFE2 expression is indicated by white arrows. *Statistically significant difference in comparison with control (*p* < 0.05). Data are shown as the mean (±s.e.m.).

We further investigated the association between p45 NFE2 and DJ-1 under oxidative stress induced by CS *in vivo*. We used lung tissue obtained from wild-type and DJ-1 KO mice exposed to 150 mg/m^3^ CS for 3 weeks. We analyzed p45 NFE2, p18 NFE2, MMP9, CD147, cathepsin B and ADAMTSL-4 expression by Western blotting. We have not observed any significant changes in these protein expression in wild type mice exposed to CS compared to control (Fig. 5A). However, we found significantly higher expression of p45 NFE2 and CD147 in DJ-1 KO mice exposed to CS in comparison with control mice (Fig. 5B). Interestingly, we also detected that CS significantly increased p45 NFE2 levels in DJ-1 KO mice compared to wild type mice (Supplementary Fig. S6). Our results suggest a compensatory function of p45 NFE2 in the absence of DJ-1. We didn’t observe changes in MMP9, ADAMTSL-4 and cathepsin B expression in lung tissue in DJ-1 KO mice. We also wanted to determine p45 NFE2 levels in murine ATII cells by immunohistofluorescence. We found higher p45 NFE2 expression in ATII cells in DJ-1 KO mice exposed to CS compared to control or wild-type mice (Fig. 5C), which correlates with our data obtained by Western blotting.

We also analyzed MMP9, CD147, cathepsin B, p45 NFE2 and ADAMTSL-4 expression by RT-PCR in lung tissue obtained from wild-type mice and DJ-1 KO mice exposed to CS. This exposure did not increase their levels in wild-type mice compared to control (Fig. 5D). However, we found significantly higher p45 NFE2 and CD147 levels in DJ-1 KO mice exposed to CS (Fig. 5E). We did not observe changes in MMP9, ADAMTSL-4 and cathepsin B levels in DJ-1 KO mice. These data correlate with our results obtained from Western blotting.

We observed co-localization of p45 NFE2 and DJ-1 in ATII cells in wild-type mice by immunohistoflurescence (Supplementary Fig. S7). We also found higher DJ-1 expression after exposure to cigarette smoke. Furthermore, we analyzed murine ATII cell apoptosis using PARP staining by immunohistofluorescence and observed higher cell apoptosis in DJ-1 KO mice than wild-type mice exposed to cigarette smoke (Supplementary Fig. S8). Our results confirm the interplay between p45 NFE2 and DJ-1 under oxidative stress induced by CS. They also suggest that induction of p45 NFE2 may compensate the absence of DJ-1.

## Discussion

Emphysema involves irreversible damage of the alveolar wall, alveolar epithelial cell death and ECM degradation^2,30^. However, there are very limited reports on isolated ATII cells obtained from patients with this disease. Therefore, here we focused on the mechanism of dysregulated antioxidant defense system in human primary ATII cells, which may contribute to an impaired proteinase-antiproteinase balance and emphysema development.

We concentrated on the cytoprotective role of p45 NFE2 against ATII cell injury induced by CS. We used ATII cells isolated from control non-smoker and smoker organ donors and patients with emphysema. Lower expression of p45 NFE2 in patients with this disease may correlate with a high ECM degradation as detected by an elevation of MMP9, CD147 and cathepsin B in ATII cells. Our results are in agreement with observations that MMP9 can alter ECM and induce progressive airspace enlargement leading to emphysema development^31–35^. It has been reported that CD147 was increased in bronchoalveolar lavage fluid obtained from COPD patients, which may suggest its modulatory role in MMP9 activation^10^. Previously published studies indicate that cathepsin B mediates human alveolar epithelial cell death stimulated by lipopolysaccharides^36^ and executing fetal ATII cell death induced by ROS generated by hyperoxia^37^. Our results suggest that cathepsin B may contribute to ATII cell injury under high oxidative stress induced by CS and in emphysema as well. ADAMTSL-4 (also known as TSRC1) belongs to metalloproteinase family however, its function remains largely unknown. We found its significantly higher expression in ATII cells and lung tissue obtained from smokers and patients with emphysema compared to non-smokers. ADAMTSL-4 expression may be related to percent parenchyma in patients with COPD and lung cancer^38^. In addition, it has been reported that ADAMTSL-4 interacted with cathepsin B in OV-90 cell line and its overexpression induced cell apoptosis^29^. Of note, the promotor region of ADAMTSL-4 has two specific DNA binding sites for a NFE2 transcription factor. This indicates that ADAMTSL-4 expression may be regulated by NFE2. Our data suggest that ADAMTSL-4 may be involved in emphysema pathogenesis, however its role remain to be further elucidated.

P45 NFE2 and NRF2 have similar DNA-binding specificities^25^. We previously reported that NRF2 has a cytoprotective activity against human and murine primary ATII and ATI-like cell injury induced by CS^19,22,23^. Here we hypothesized that p45 NFE2 also provides cytoprotection against oxidative stress in ATII cells. We found significantly lower expression of p45 NFE2 in ATII cells isolated from patients with emphysema in comparison with control smokers and non-smokers by Western blotting. We further studied the functional role of NFE2 *in vitro*. We found that NFE2 knockdown increased cell death induced by CSE as detected by Annexin V and PI staining and flow cytometry analysis. Our findings are supported by observations that p45 NFE2 was identified as a regulator of cytoprotective genes in megakaryocytes^25^. It was reported that low ROS levels in cells are maintained mainly by NRF2 induction. However, P45 NFE2 competes with NRF2 and can maintain moderate expression of the cytoprotective genes. Moreover, p45 NFE2 KO mice have decreased expression of genes involved in a response to oxidative stress^39^. This indicates that p45 NFE2 has a cytoprotective function; however it has a weaker activity than NRF2. This is in agreement with our previous studies showing strong activation of antioxidant defense system regulated by NRF2 in ATII cells in response to oxidative stress induced by CSE and influenza virus^21,22,40^. Furthermore, it has been reported that NFE2 activity can be affected by posttranslational modifications such as phosphorylation^41^. Therefore, we also analyzed p45 NFE2 phosphorylation to further determine its function in ATII cells. We found statistically significant decreased phosphorylation of tyrosine within p45 NFE2 in ATII cells isolated from control smokers. Our results are in agreement with previous observations showing that inhibition of tyrosine phosphorylation augments the enhancer activity of the NFE2 function^42^. This suggests its cytoprotective role against ATII cell injury in smokers.

**Figure 6:**
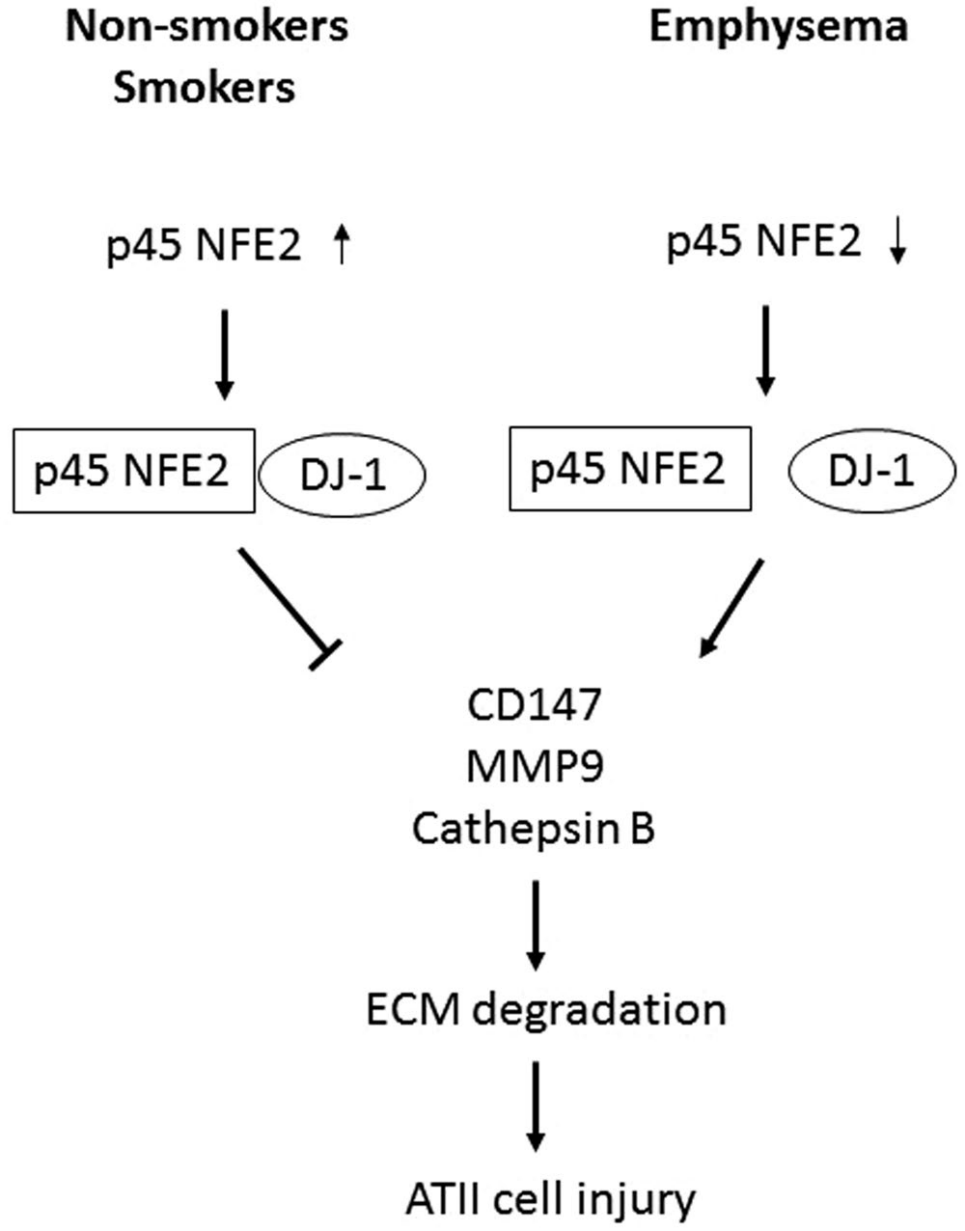
The role of p45 NFE in ATII cells in non-smokers, smokers and emphysema patients.

We have previously shown that DJ-1 modulates expression of NRF2 and downstream cytoprotective genes in human primary ATII cells exposed to CSE *in vitro* or isolated from heavy smokers^19^. Therefore, here we wanted to checked for the first time the association between DJ-1 and p45 NFE2. We found the lowest interaction between p45 NFE2 and DJ-1 in ATII cells isolated from emphysema patients, which correlates with decreased p45 NFE2 expression in these individuals. To further study the functional role of p45 NFE2 and DJ-1 interaction, we used lung tissue obtained from wild-type and DJ-1 KO mice exposed to CS. First, we found that CS significantly increased p45 NFE2 levels in DJ-1 KO mice after 3 weeks of exposure. This suggests that the absence of DJ-1 affects p45 NFE2 expression under oxidative stress. Second, p45 NFE2 levels in DJ-1 KO mice exposed to CS were significantly higher compared to wild-type mice, which indicates that p45 NFE2 may compensate the lack of cytoprotective DJ-1. Third, we also found increased expression of CD147 in DJ-1 KO mice. The functional link between CD147 and NFE2 has been already reported by Schultz *et al*.^43^ in vascular inflammation *in vivo*. Fourth, we didn’t observe differences in MMP9, cathepsin B and ADAMTSL-4 levels in wild-type and DJ-1 KO mice. This indicates that higher concentration and/or longer exposure to CS may be required for their induction. In summary, our results suggest that oxidative stress by CS induces p45 NFE2 expression, which activates a cytoprotection against cell injury (Fig. 6). P45 NFE2 downregulation in human primary ATII cells may contribute to emphysema pathogenesis. Targeting p45 NFE2 may provide a potential therapeutic strategy against oxidative stress-induced lung injury.

## Methods

### ATII cell isolation from control organ donors and patients with emphysema

We obtained tissue from de-identified control non-smoker and smoker organ donors whose lungs were not suitable for transplantation and donated for medical research from the Gift of Life Donor Program. We selected donors with a clinical history and x-ray that did not indicate infection, reasonable lung function with a PaO_2_/FIO_2_ ratio of > 250, and limited time on a ventilator. Non-smokers were individuals who never smoked and smokers smoked 10–20 cigarettes per day for at least 3 years. We also obtained tissue from patients with emphysema (GOLD IV) from lung volume reduction surgery through Temple Biobank (Temple University, Philadelphia, PA). ATII cells were isolated as we previously described (N = 14 per group)^23^. Briefly, after elastase (Worthington, Lakewood, NJ) instillation, lung was minced and the cells were filtrated and purified by centrifugation on density gradients made of Optiprep (Accurate Chemical Scientific Corp., Westbury, NY) followed by negative selection with CD14-coated magnetic beads (Dynal Biotech ASA, Oslo, Norway) and binding to IgG-coated (Sigma Chemicals Inc., St. Louis, MO) dishes. The purity of isolated ATII cells was 85% as determined by staining for cytokeratin CAM 5.2 (Dako, Carpinteria, CA, data not shown)^23^. All experiments were performed in accordance with relevant guidelines and regulations. The study was performed in accordance with the Declaration of Helsinki and was approved by the Institutional Review Boards at Partners Healthcare and the Committee for the Protection of Human Subjects at Temple University.

### Western Blotting and Immunoprecipitation

GAPDH and NRF2 antibodies were obtained from Abcam (Cambridge, MA) and β-actin was purchased from Sigma-Aldrich (St. Louis, MO). The following antibodies were obtained from Santa Cruz Biotechnology (Santa Cruz, CA): MMP9, CD147, ADAMTSL-4, p45 NFE2, p18 NFE2, DJ-1, phosphoserine, phosphothreonine and phosphotyrosine. Lung tissue used for Western blotting was homogenized in Tissue PE-LB buffer (G-Biosciences, Saint Louis, MO) and ATII and A549 cells were lysed in Mammalian Cell PE buffer (G-Biosciences) with protease and phosphatase inhibitors (Gold Biotechnology, St. Louis, MO). Western blotting was performed as we previously described^19^. Briefly, cell or tissue lysates were analyzed for protein expression using polyacrylamide gradient gels (8–16%; Thermo Fisher Scientific, Waltham, MA). Nitrocellulose membrane was used for protein transfer, followed by blocking with 5% bovine serum albumin and incubation with primary antibodies overnight at 4 °C. On the following day, membranes were incubated for 1 h with horseradish peroxidase (HRP)-conjugated AffiniPure donkey anti-rabbit IgG or HRP-conjugated AffiniPure donkey anti-mouse IgG purchased from Jackson ImmunoResearch (West Grove, PA). The blots were developed using Luminata Forte Western HRP Substrate (Millipore, Billerica, MA) for chemiluminescent detection of protein expression. The density of bands was quantified using ImageJ and NIH Image 1.62 software (Bethesda, MD). For immunoprecipitation, the lung tissue was homogenized and ATII cells were lysed as described above and p45 NFE2, p18 NFE2 or DJ-1 antibodies were added to the lysate overnight. The immunoprecipitates were collected by incubating with Protein A/G Plus Agarose beads (Santa Cruz Biotechnology, CA) for 2 h. The beads bearing the immunoprecipitates were washed three times with PBS and used for Western blotting as described above.

### RT-PCR

RT-PCR was used to analyze human and murine MMP9, CD147, ADAMTSL-4, cathepsin B and p45 NFE2 mRNA expression. Gene-specific primers were retrieved from PrimerBank (http://pgamgh.harvard.edu/primerbank/) and ordered from Invitrogen (Waltham, MA). The sequences of the primers are provided in Supplementary Table S1. ATII cells were lysed and lung tissue was homogenized in RLT buffer (Qiagen, Germantown, MD). Total RNA was isolated from these samples using Quick-RNA MiniPrep (Zymo Research, Irvine, CA). A total of 1 µg total RNA was reverse transcribed into cDNA using the SuperScript IV First-Strand Synthesis System (Thermo Fisher Scientific, Waltham, MA) according to the manufacturer’s recommendations. mRNA expression was determined by RT-PCR using the SYBR Green Master Mix (Applied Biosystems, Foster City, CA). Thermal cycle conditions were initial denaturation at 95 °C for 10 min followed by 45 cycles of 95 °C for 15 s, 58 °C for 60 s and 68 °C for 20 s. Results were analyzed using the ΔCt method.

### Immunohistofluorescence

Human and murine lung tissue sections were fixed in 4% paraformaldehyde (Electron Microscopy Sciences, Hatfield, PA) and embedded in paraffin. Sections were deparaffinized and hydrated followed by antigen retrieval by boiling in 0.01 M citrate buffer (pH 6.0) for 10 min. Lung sections were blocked with 3% normal donkey serum (Sigma-Aldrich, St. Louis, MO) in PBS for 20 min followed by incubation with antibodies: anti-SP-A (Santa Cruz), PARP (Cell Signaling), p45 NFE2 or DJ-1 overnight. The secondary antibodies, Alexa Fluor 594 IgG, Alexa Fluor 488 IgG and Alexa Fluor 647 IgG (Thermo Fisher Scientific, Waltham, MA) were applied for 1 h. Sections were mounted with Fluoroshield Mounting Medium containing DAPI (Abcam, Cambridge, MA) and analyzed using Zeiss Axio Imager Confocal Fluorescent Microscopy.

### NFE2 knockdown

A549 cells (ATCC, Manassas, VA) were cultured in DMEM supplemented with 10% FBS, penicillin (100 IU/ml) and streptomycin (100 μg/ml). Cells with 80% confluency were transfected with 100 nM NFE2 siRNA (Santa Cruz, CA) or NT (non-targeting) siRNA: 5′ UAGCGACUAAACACAUCAAUU 3′ and 3′ UUAUCGCUGAUUUGUGUAGUU 5′. We used Lipofectamine 3000 (ThermoFisher, Waltham, MA) for cell transfection according to the manufacturer’s protocol. Briefly, Lipofectamine 3000 and NFE2 siRNA or NT siRNA were mixed in Opti-MEM (ThermoFisher, Waltham, MA) and incubated for 5 min. This mixture was added to A549 cells for 48 h and after that replaced with DMEM with FBS and antibiotics followed by treatment with cigarette smoke extract as described below. Knockdown of p45 NFE2 was determined by Western blotting.

### Preparation of CSE

The cigarette smoke extract (CSE) was prepared using one 3R4F cigarette without filter (Kentucky Tobacco Research & Development Center, Lexington, KY) as we previously described^21^. Briefly, we prepared 100% CSE in 12.5 ml DMEM without FBS using a peristaltic pump (Mannostat 72-310-000; Barrington, IL) and filtered through 0.22μm filter. The CSE was diluted to 40% and used immediately for A549 cell treatment for 24 h.

### Annexin V/PI staining

A549 cells with NFE2 knockdown were treated with CSE as described above and cell death was analyzed by flow cytometry analysis. A549 cells were resuspended in Annexin V binding buffer and stained with 5 μl Annexin V conjugated to Alexa Fluor 488 (Thermo Fisher Scientific, Waltham, MA) and 1 μg/ml propidium iodide (PI) at room temperature for 5 min. Cell death was analyzed using LSR-II flow cytometer (BD Biosciences, San Jose, CA) and FlowJo (TreeStar).

### Mice Exposure to Cigarette Smoke

We used wild-type C57BL/6 mice and DJ-1 KO mice (N = 4 per group) obtained from The Jackson Laboratory (Bar Harbor, ME). Mouse colonies were maintained at Temple University. All mice were fed ad libitum and housed in an Institutional Animal Care and Use Committee (IACUC)-accredited facility in individually ventilated cages. Animal care, handling and experimental procedures were carried out in accordance with a protocol approved by the IACUC of Temple University. We exposed 7-week-old mice to CS generated from Kentucky reference cigarette 3R4F (University of Kentucky) for 2 h per day for 3 weeks using a Teague TE-10 smoking system (Teague Enterprises, Woodland, CA, USA). The average particulate matter was 150 mg/m^3^ and carbon monoxide levels were <300 ppm.

### Statistics

We used t-test to determine the statistically significant difference (*p* < 0.05). Data are shown here as the mean ± s.e.m. from at least three independent experiments.

### Data availability

All data generated or analyzed during this study are included in this published article and its Supplementary Information files.

## Electronic supplementary material

Supplementary information
