# Supplementary material for: The cytoprotective role of DJ-1 and p45 NFE2 against human primary alveolar type II cell injury and emphysema
Source: Sci Rep. 2018 Feb 23;8:3555. doi: 10.1038/s41598-018-21790-3 (PMC5824795; doi:10.1038/s41598-018-21790-3)
Supplement: Supplementary file 1 — Supplementary information [file 41598_2018_21790_MOESM1_ESM.pdf]

Supplementary Information

The cytoprotective role of DJ-1 and p45 NFE2 against human primary ATII cell injury and emphysema

Li Hui Tan[1,2], Karim Bahmed[1,2], Chih-Ru Lin[1,2], Nathaniel Marchetti[1,2], Sudhir Bolla[1,2], Gerard J. Criner[1,2], Steven Kelsen[1,2], Muniswamy Madesh[3], Beata Kosmider[1,2,4,*]

[1] Department of Thoracic Medicine and Surgery, Temple University, Philadelphia, PA 19140

[2] Center for Inflammation, Translational and Clinical Lung Research, Temple University, Philadelphia, PA 19140

[3] Medical Genetics and Molecular Biochemistry, Temple University, Philadelphia, PA 19140

[4] Department of Physiology, Temple University, Philadelphia, PA 19140

* Correspondence:

Beata Kosmider, Ph.D.

Department of Physiology

Department of Thoracic Medicine and Surgery

Center for Inflammation, Translational and Clinical Lung Research

Temple University

3500 N. Broad Street

Philadelphia, PA 19140

E-mail: beata.kosmider@temple.edu

Supplementary Table S1. Sequences of the primers used to analyze human (A) and murine (B) gene expression by RT-PCR.

A

**Human Gene**	**Sequence**	
MMP9	Fw	3’ GGGACGCAGACATCGTCATC 5’
MMP9	Rev	3’ TCGTCATCGTCGAAATGGGC 5’
CD147	Fw	3’ GAAGTCGTCAGAACACATCAACG 5’
CD147	Rev	3’ TTCCGGCGCTTCTCGTAGA 5’
ADAMTSL-4	Fw	3’ CATCAAGCCAGGAATGTTCGG 5’
ADAMTSL-4	Rev	3’ AGGGGACGGAATAGCCTCTTC 5’
Cathepsin B	Fw	3’ AGAGTTATGTTTACCGAGGACCT 5’
Cathepsin B	Rev	3’ GATGCAGATCCGTCAGAGA 5’
p45 NFE2	Fw	3’ CCACCACCCACAACTTACTG 5’
p45 NFE2	Rev	3’ GAGGGCTAAGGGGTCTTGGA 5’
GAPDH	Fw	3’ GGAGCGAGATCCCTCCAAAAT 5’
GAPDH	Rev	3’ GGCTGTTGTCATACTTCTCATGG 5’

B

**Murine Gene**	**Sequence**	
MMP9	Fw	3’ GGACCCGAAGCGGACATTG 5’
MMP9	Rev	3’ CGTCGTCGAAATGGGCATCT 5’
CD147	Fw	3’ CCTGCATACGAAGTACATAGTGG 5’
CD147	Rev	3’ TGATTTCTTTCCGACCTTGATCC 5’
ADAMTSL-4	Fw	3’ GAACCCCGTTCTCACTCAGC 5’
ADAMTSL-4	Rev	3’ TGGAAAGACCTACTGTCTCCAAA 5’
Cathepsin B	Fw	3’ TTGCGTTCGGTGAGGACATAG 5’
Cathepsin B	Rev	3’ GCAGGAGCCCTGGTCTCTA 5’
p45 NFE2	Fw	3’ TCCTCAGCAGAACAGGACAG 5’
p45 NFE2	Rev	3’ GGCTCAAAAGATGTCTCACTTGG 5’
GAPDH	Fw	3’ CATGGCCTTCCGTGTTCCT 5’
GAPDH	Rev	3’ CCTGCTTCACCACCTTCTT 5’

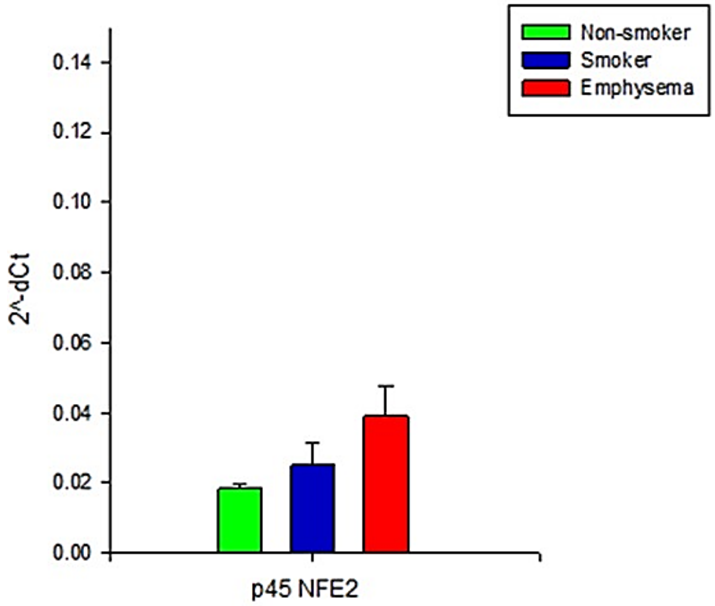

Supplementary Fig. S1 P45 NFE2 levels in ATII cells isolated from non-smokers, smokers and emphysema patients by RT-PCR. Data are shown as the mean (±s.e.m.).

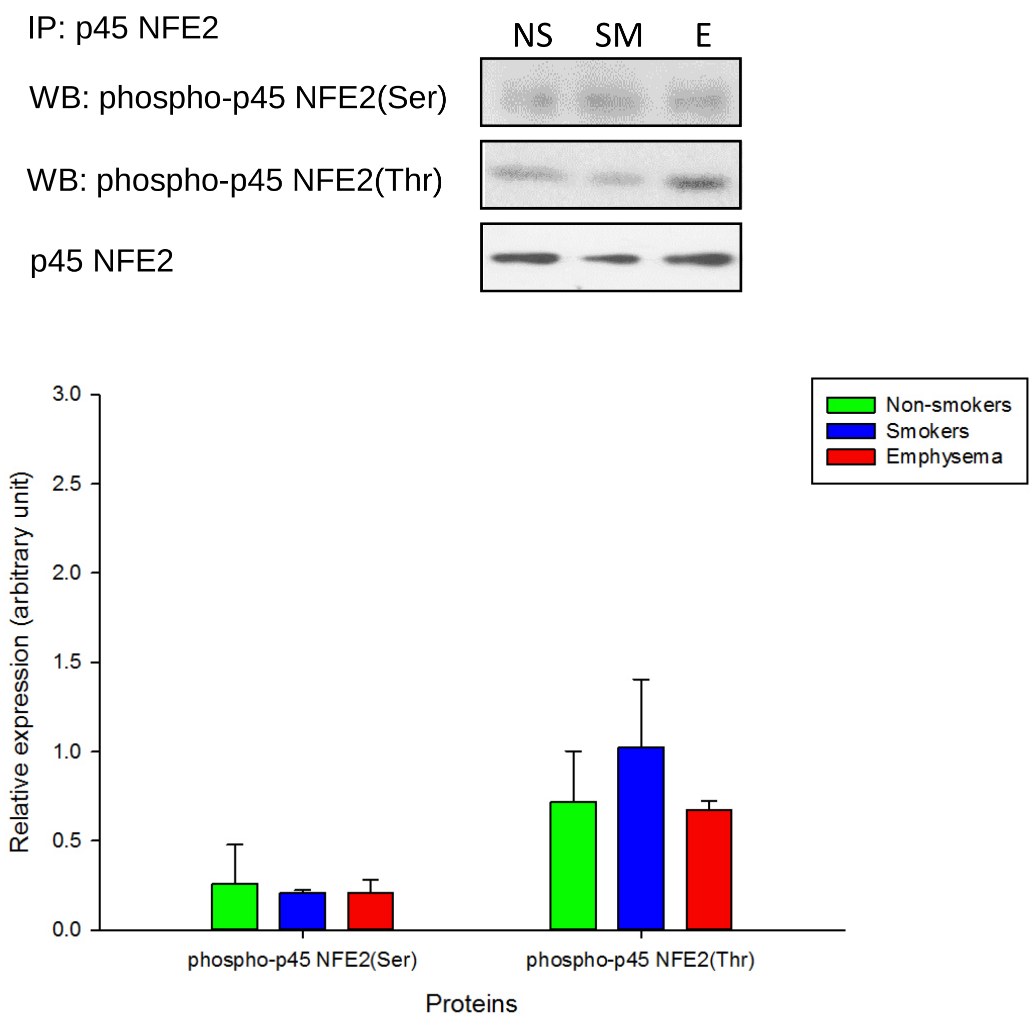

Supplementary Figure S2. p45 NFE2 phosphorylation in human lung tissue obtained from control non-smokers (NS), smokers (SM) and patients with emphysema (E). p45 NFE2 was immunoprecipitated followed by Western blotting analysis to determine serine and threonine phosphorylation. Densitometric quantification is also shown. Data are shown as the mean (±s.e.m.).

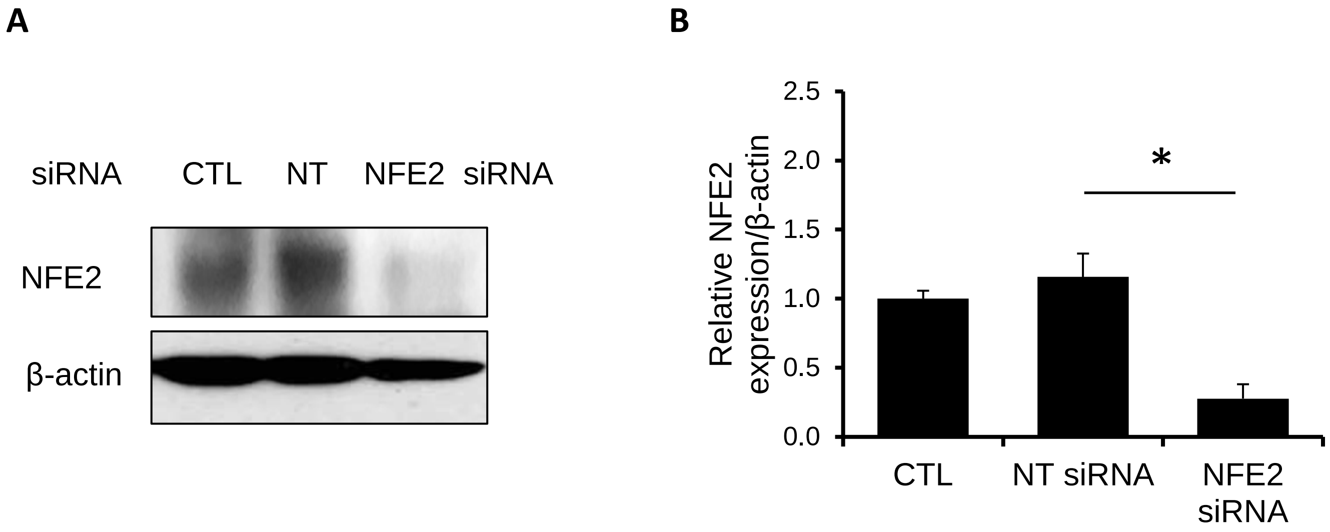

Supplementary Figure S3. NFE2 knockdown in A549 cells. A549 cells were transfected with 100 nM NFE2 siRNA or non-targeting (NT) siRNA for 48h and cell lysates were subjected to Western blot (A). CTL-control. Densitometric analysis is also shown (B). * Statistically significant difference ($p<0.05$). Data are presented as the mean (±s.e.m.).

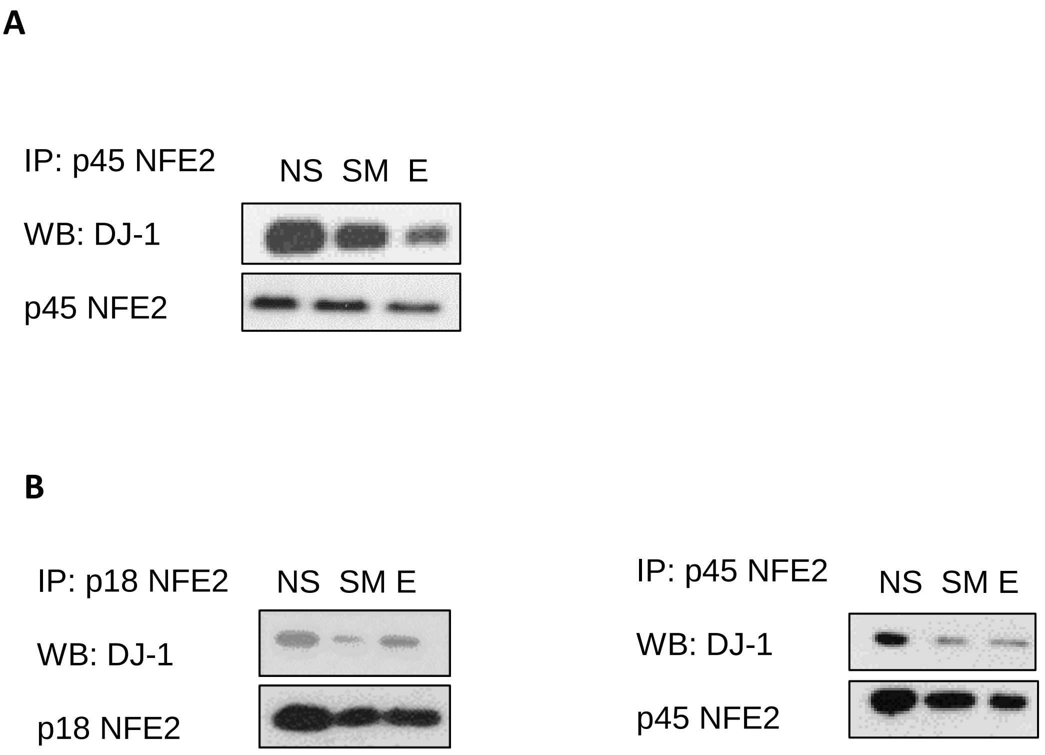

Supplementary Figure S4. p45 NFE2 interaction with DJ-1 in ATII cells and lung tissue obtained from non-smokers (NS), smokers (SM) and patients with emphysema (E). p45 NFE2 was co-immunoprecipitated in freshly isolated ATII cells (**A**) and p45 NFE2 or p18 NFE2 were co-immunoprecipitated in lung tissue (**B**). Western blotting analysis was used to determine their interaction with DJ-1 as described in Materials and Methods (N=3).

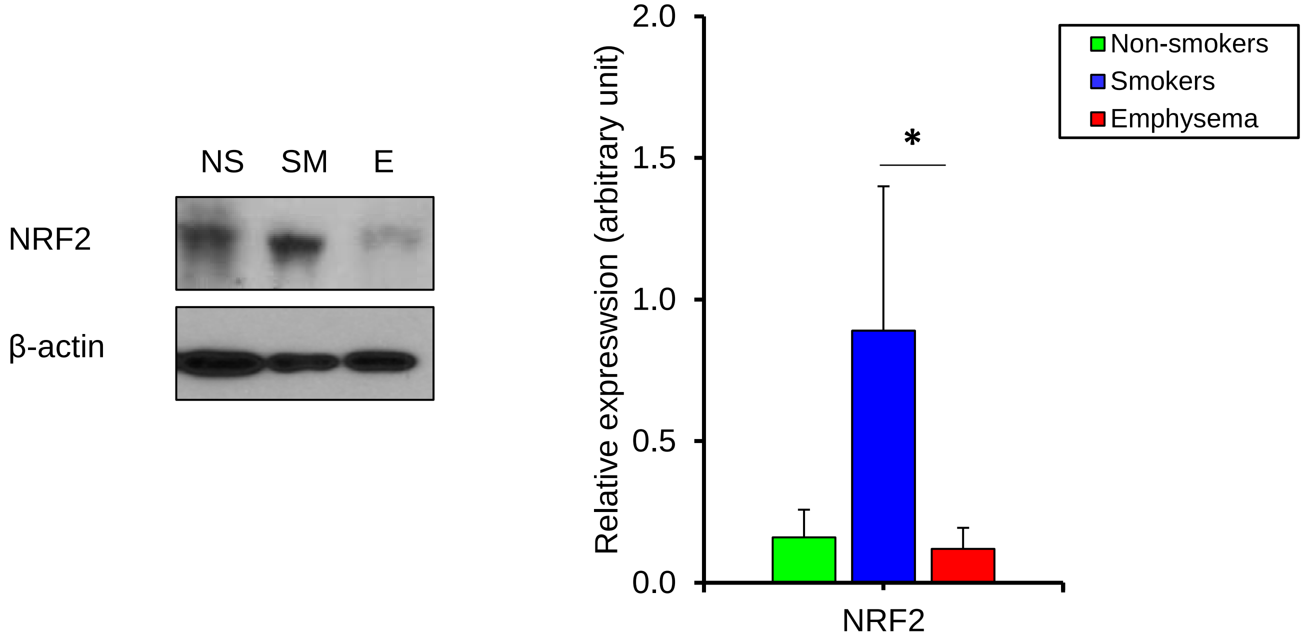

Supplementary Figure S5. NRF2 expression in ATII cells obtained from non-smokers (NS), smokers (SM) and patients with emphysema (E). Protein levels were analyzed by Western blotting. Densitometric analysis and statistically significant difference ($p<0.05$, N=3) is shown. Data are shown as the mean (±s.e.m.).

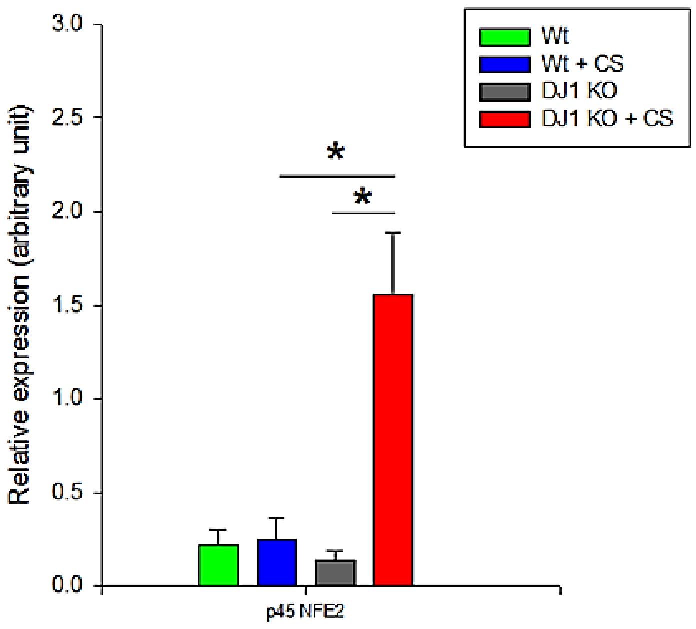

Supplementary Figure S6. Comparison of p45 NFE2 expression in wild-type and DJ-1 KO mice. Mice were exposed to 150 mg/m^3 cigarette smoke (CS) for 2h/day for 3 weeks as described in Materials and Methods section. Protein levels were analyzed in lung tissue by Western blotting as shown in Figure 5. Relative expression and statistically significant difference (* $p<0.05$) is shown. Data are presented as the mean (±s.e.m.).

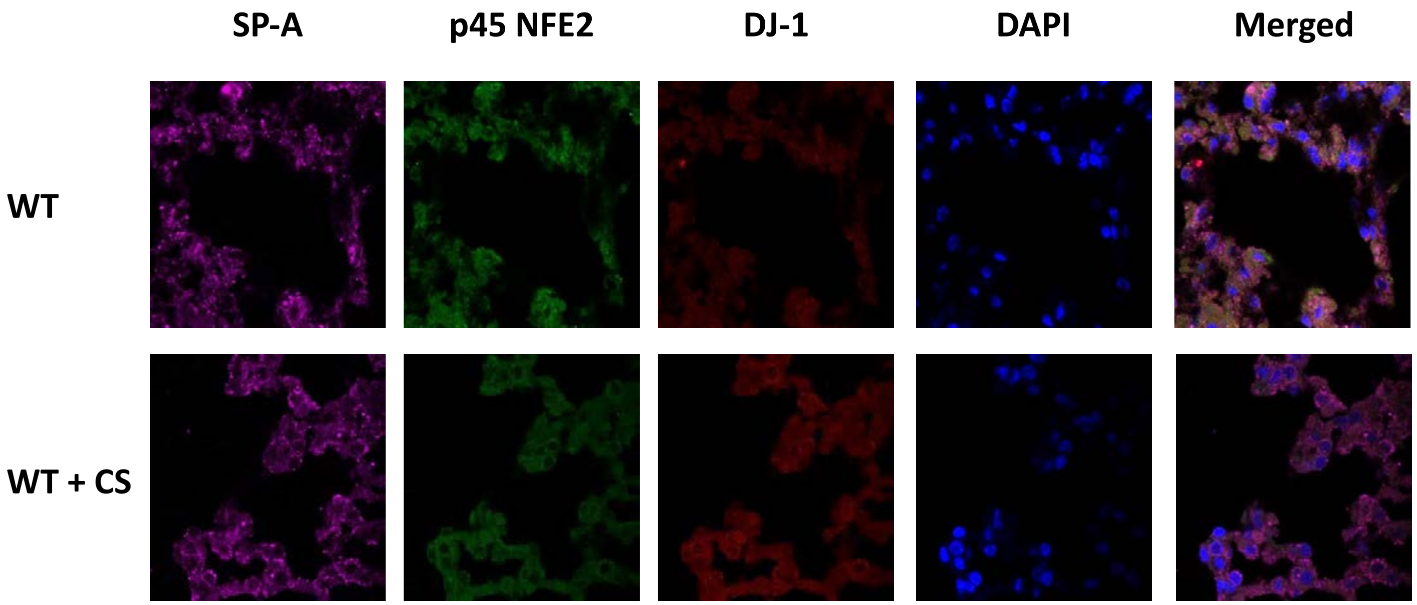

Supplementary Figure S7. P45 NFE2 and DJ-1 expression in wild-type mice. Mice were exposed to 150 mg/m3 cigarette smoke (CS) for 2h/day for 3 weeks as described in Materials and Methods. Representative p45 NFE2 (green) and DJ-1 (red) expression in ATII cells identified using SP-A antibody (violet) in murine lung tissue by immunohistofluorescence. Cell nuclei were stained with DAPI (blue; N = 3).

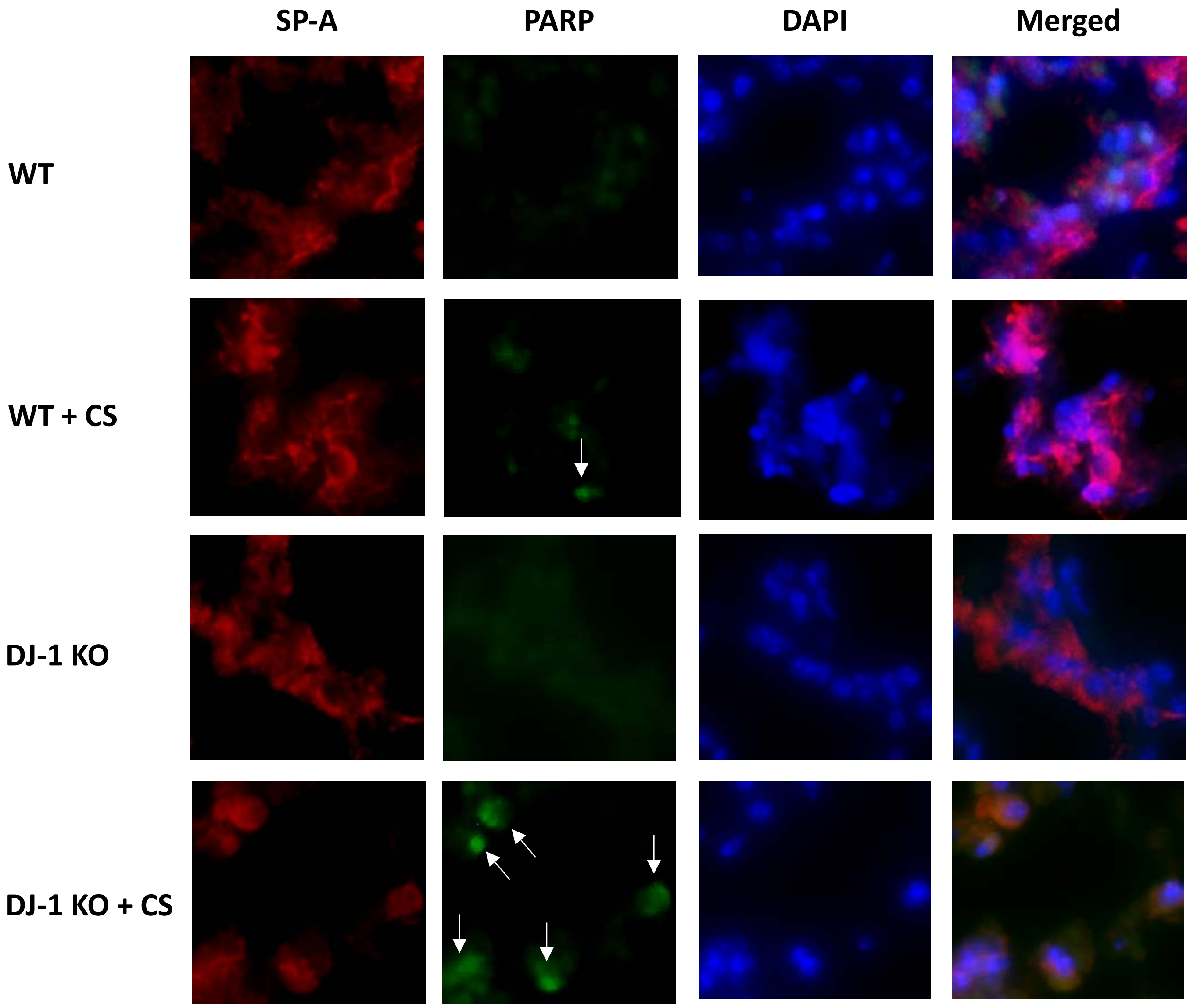

Supplementary Figure S8. Apoptotic ATII cells in DJ-1 KO mice exposed to cigarette smoke. Wild-type and DJ-1 KO mice were exposed to 150 mg/m3 cigarette smoke (CS) for 2h/day for 3 weeks as described in Materials and Methods. Representative PARP (green) expression in ATII cells identified using SP-A antibody (red) in murine lung tissue by immunohistofluorescence. White arrows indicate apoptotic ATII cells. Cell nuclei were stained with DAPI (blue; N = 3).
